# Acceptance of a COVID-19 Vaccine in Japan during the COVID-19 Pandemic

**DOI:** 10.3390/vaccines9030210

**Published:** 2021-03-03

**Authors:** Masaki Machida, Itaru Nakamura, Takako Kojima, Reiko Saito, Tomoki Nakaya, Tomoya Hanibuchi, Tomoko Takamiya, Yuko Odagiri, Noritoshi Fukushima, Hiroyuki Kikuchi, Shiho Amagasa, Hidehiro Watanabe, Shigeru Inoue

**Affiliations:** 1 Department of Preventive Medicine and Public Health, Tokyo Medical University, Tokyo 160-8402, Japan; machida@tokyo-med.ac.jp (M.M.); takamiya@tokyo-med.ac.jp (T.T.); odagiri@tokyo-med.ac.jp (Y.O.); fukufuku@tokyo-med.ac.jp (N.F.); kikuchih@tokyo-med.ac.jp (H.K.); amagasa@tokyo-med.ac.jp (S.A.); 2 Department of Infection Prevention and Control, Tokyo Medical University Hospital, Tokyo 160-0023, Japan; task300@tokyo-med.ac.jp (I.N.); hw-nabe4@tokyo-med.ac.jp (H.W.); 3 Department of International Medical Communications, Tokyo Medical University, Tokyo 160-0023, Japan; tkojima@tokyo-med.ac.jp; 4 Division of International Health (Public Health), Graduate School of Medical and Dental Sciences, Niigata University, Niigata 951-8510, Japan; jasmine@med.niigata-u.ac.jp; 5 Graduate School of Environmental Studies, Tohoku University, Miyagi 980-0845, Japan; tomoki.nakaya.c8@tohoku.ac.jp (T.N.); info@hanibuchi.com (T.H.)

**Keywords:** COVID-19, vaccine, public health, epidemiology, vaccine hesitancy

## Abstract

Vaccination could be a key protective measure against coronavirus disease 2019 (COVID-19), and it is important to understand the acceptability of the COVID-19 vaccine among the general public. However, there is no study on the acceptance of a COVID-19 vaccine in Japan. Therefore, this study aimed to describe the COVID-19 vaccine acceptance and hesitancy situation in Japan and assess the factors associated with such issues. This was a cross-sectional study based on an internet survey completed by 2956 people. Participants were asked to indicate how likely they were to get vaccinated for COVID-19. In addition, the participants responded to questions regarding sociodemographic factors, attitudes, and beliefs regarding COVID-19 infection and vaccination. The proportion of participants with a high likelihood of getting a COVID-19 vaccine was 62.1%. Multiple logistic regression analysis showed that vaccine acceptance was lower among several sociodemographic groups, such as women, adults aged 20–49 years, and those with a low-income level. Several psychological factors, especially the perceived effectiveness of the COVID-19 vaccine, and willingness to protect others by getting oneself vaccinated, were associated with vaccine acceptance. Our results indicate that the perceived effectiveness of the vaccine and willingness to protect others may play an important role in the acceptance of the COVID-19 vaccine.

## 1. Introduction

The coronavirus disease 2019 (COVID-19) pandemic is a public health threat [1]. Various COVID-19 pandemic mitigation strategies, such as large-scale physical distancing measures and movement restrictions, often referred to as ‘lockdowns’, have been implemented in various countries [2,3], but the pandemic is still ongoing despite such efforts. Although implementation of personal protective measures by ordinary citizens is key to managing the spread of this infectious disease, vaccination could be a key protective measure against COVID-19 [4]. The development and production of the COVID-19 vaccine accelerated to a speed that has been unheard of. Various initiatives, such as large-scale funding and industrial-scale manufacturing of the vaccine well before the demonstration of vaccine efficacy and safety and human challenge study, have been considered and implemented [5,6,7]. As of February 2021, approximately 70 vaccines have been tested in clinical trials on humans, and 20 in phase III clinical trials [8,9]. Furthermore, several vaccines have already been approved in some countries, and vaccination has started [10,11]. In order to plan adequate COVID-19 vaccine uptake among the general public, understanding the public’s concerns regarding the COVID-19 vaccine is essential [12,13]. As with previous studies on vaccine hesitancy for vaccines [13], studies on COVID-19 vaccine acceptance have demonstrated that the acceptability of the vaccine differs depending on sociodemographic factors, such as race and educational level, as well as attitudes and beliefs regarding COVID-19 infection and vaccination [14,15,16,17,18,19]. A global survey, which included 19 countries, also reported that responses in different countries show that COVID-19 vaccine acceptance has high heterogeneity [20]; therefore, it is important to clarify the acceptance of a vaccine in each country or region. In Japan, cost-free vaccination for COVID-19 is scheduled to begin in the spring of 2021 for the general public [21]. However, to the best of our knowledge, there is currently no study that highlights COVID-19 vaccine acceptance in Japan. Japan has one of the lowest vaccine confidence indexes in the world [22], which may be due to an incident in 2013, where the Japanese Ministry of Health, Labor, and Welfare suspended proactive recommendation of the human papillomavirus vaccine because of safety concerns raised by the general public [23,24]. Naturally, there are concerns regarding COVID-19 vaccine acceptance in the country.

Therefore, the aim of this study was to describe and evaluate the COVID-19 vaccine acceptance and hesitancy situation in Japan, and to assess any associated factors.

## 2. Materials and Methods

### 2.1. Study Sample and Data Collection

This was a cross-sectional study conducted through an internet-based survey. The survey was conducted between 14 January 2021 and 18 January 2021. At the time of the survey, the number of confirmed COVID-19 cases in Japan had significantly increased, and a state of emergency was declared for the Tokyo metropolitan area (i.e., Tokyo, Kanagawa, Saitama, and Chiba prefecture) by the Japanese government on January 7, 2021, for the second time since April 2020 [25]. The study participants were recruited from the registrants of a Japanese Internet research service company called MyVoice Communication, Inc., which had approximately 1.12 million registered participants as of February 2020. We aimed to collect data from 3000 20 to 79-year-old men and women from all regions of Japan. Quota sampling based on age, sex, and residential area was used to ensure that the sample was broadly representative of the general population of Japan. We stratified the 3000 participants according to sex, age (five-year age groups), and residential area (i.e., Hokkaido, Tohoku, Kanto, Chubu, Kinki, Chugoku, Shikoku, and Kyushu regions), and set a target number of respondents for each group.

The Internet research service company randomly chose potential respondents from the registered participants (*n* = 13,191) and invited them to participate in the survey by email on 14 January 2021. The questionnaires were placed in a secured section of a website, and potential respondents received a specific URL in their invitation email. When the number of participants who responded to the questionnaire voluntarily reached the target number of respondents for each group, responses were no longer accepted for that group. The survey was concluded on 18 January 2021, when the target number of respondents was reached for all groups. Reward points valued at 80 yen were provided as incentives for participation (approximately 0.8 U.S. dollars as of January 2021). This study was approved by the Ethics Committee of Tokyo Medical University, Tokyo, Japan (No: T2019-0234). Informed consent was obtained from all respondents.

### 2.2. Assessment of Participants’ Likelihood of Getting a COVID-19 Vaccine and Their Attitudes and Beliefs Regarding COVID-19 Vaccination

We asked the participants to indicate how likely they were to get vaccinated for COVID-19, once a vaccine is available to the public. With reference to a previous study, five response options were provided: very unlikely, somewhat unlikely, somewhat likely, very likely, and unsure [18].

To measure attitudes and beliefs regarding COVID-19 vaccination, we used survey items from the COVID-19 Snapshot Monitoring questionnaire, which was developed to monitor public perceptions of COVID-19 based on validated questionnaires [26,27]. The survey items were translated into Japanese, which was followed by a back translation to confirm accuracy of the initial translation. With reference to previous studies [12,14,15,16,17,28], we asked the participants to provide responses for the following items: perceived likelihood of becoming infected with COVID-19 in the future, perceived severity of a COVID-19 infection, perceived effectiveness of a COVID-19 vaccine, and willingness to protect others by getting oneself vaccinated. We also assessed the significance of identified factors (three items: vaccine safety, vaccine accessibility, and doctor’s recommendation) and their influence on respondents’ decision-making regarding vaccination. The range of possible responses for these items were coded 1–7. We coded each attitude and belief variable so that higher values indicated greater levels of that construct. Supplementary material shows the actual questions and response options translated from Japanese to English.

### 2.3. Assessment of Sociodemographic Factors

Participants reported their sex, age, underlying diseases, including heart diseases, respiratory diseases, kidney diseases, diabetes, and hypertension (yes or no), marital status (married or not married), employment status (working or not working), residential area (prefectures, 47 items), and living arrangement (alone or with others).

The research company provided categorized data on educational attainment (university graduate level or above) and annual personal income level (less than 2 million yen (approximately 19,000 USD), 2–4 million yen (19,000–38,000 USD), 4–6 million yen (38,000–57,000 USD), 6 million yen or more (57,000 USD).

### 2.4. Statistical Analysis

Regarding participants’ likelihood of getting a COVID-19 vaccine, if a participant responded “very likely” or “somewhat likely”, it was determined that the participant had a high likelihood of getting a COVID-19 vaccine [18]. We clarified the proportion of participants with a high likelihood of getting a COVID-19 vaccine.

Regarding attitudes and beliefs about COVID-19 infection and COVID-19 vaccination, if a participant responded with 1–3, 4, or 5–7 on the scale, the level of that construct was defined as low, middle, or high, respectively. Participants’ characteristics, attitudes, and beliefs were compared using a chi-square test. Participants who had a high likelihood of getting a COVID-19 vaccine were compared with those who did not.

Multiple logistic regression analysis was performed to clarify the association between each factor and high likelihood of getting a COVID-19 vaccine. The dependent variable was set as a dichotomous variable coded as “1” if the participant had a high likelihood of getting a COVID-19 vaccine, and “zero” otherwise. In model 1, the independent variables were sex, age (20–49/50–64/≥65 years), underlying diseases, marital status, employment status, residential area (Tokyo metropolitan area/other areas), living arrangement, educational attainment, and annual personal income. In model 2, the independent variables were the factors in model 1, plus perceived likelihood of becoming infected with COVID-19 in the future, perceived severity of a COVID-19 infection, perceived effectiveness of a COVID-19 vaccine, willingness to protect others by getting oneself vaccinated, and the significance of identified factors influencing respondents’ decision-making regarding vaccination (three items). The independent variables included factors known to influence vaccine acceptance from previous studies as well as those measured in this study [15,16,17,18,19]. Statistical analyses were performed using IBM SPSS Statistics for Windows, version 27 (IBM Japan, Tokyo, Japan). Two-sided *p*-values less than 0.05 were considered statistically significant.

## 3. Results

Of the 3000 participants selected for this study, 44 participants with incomplete data provided by the survey company were excluded from the analysis. Therefore, the analysis set consisted of 2956 participants (Table 1). The proportion of participants with a high likelihood of getting a COVID-19 vaccine was 62.1%. A significantly lower proportion of participants who were women, aged 20–49 years, with no underlying diseases, not married, with a low educational level, and a low annual personal income had a high likelihood of getting a COVID-19 vaccine compared with participants who were men, aged 65 years and older, with underlying diseases, married, with a high educational level, and a high annual personal income, respectively.

Table 2 shows the attitudes and beliefs of the participants regarding COVID-19 infection and COVID-19 vaccination. For all survey items, the proportion of participants with a high likelihood of getting a COVID-19 vaccine was significantly higher among those with a high level of that specific construct, compared with participants who had a low level of that construct.

Table 3 shows the results of logistic regression analysis. Regarding sociodemographic factors, having underlying diseases was a significant factor for having a high likelihood of getting a COVID-19 vaccine (having underlying diseases; odds ratio (OR): 1.32, 95% confidence interval (95% CI): 1.06–1.65), whereas being a woman, being aged 20–49 years, and having a low annual personal income level were significant factors for being unlikely to get a COVID-19 vaccine (women; OR: 0.67, 95% CI: 0.55–0.83, 20–49 years old; OR: 0.59, 95% CI: 0.45–0.77, <2 million yen; OR: 0.67, 95% CI: 0.47–0.95). Regarding attitudes and beliefs about COVID-19 vaccination, possessing high levels of each construct in many survey items was a significant factor for having a high likelihood of getting a COVID-19 vaccine; however, the result for the ‘doctor’s recommendation’ item was not significant. The ORs for having a high level of perceived effectiveness of a COVID-19 vaccine and willingness to protect others by getting oneself vaccinated were particularly high (having a high level of perceived effectiveness of a COVID-19 vaccine; OR: 9.15, 95% CI: 6.69–12.51) (having a high level of willingness to protect others by getting oneself vaccinated; OR: 3.51, 95% CI: 2.75–4.48).

## 4. Discussion

In this study, we aimed to determine the current COVID-19 vaccine acceptability and hesitancy situation in Japan and the factors associated with these issues. We found that nearly 62.1% of adults in Japan would be willing to receive a COVID-19 vaccine if one becomes available. Some sociodemographic factors, such as sex, age, and income level, were associated with vaccine acceptance, in addition to some psychological factors, especially the perceived effectiveness of a COVID-19 vaccine, and willingness to protect others by getting oneself vaccinated. Our findings represent one of the first estimates of the acceptance of a COVID-19 vaccine in Japan that can be used to plan COVID-19 vaccine uptake among the general public.

Studies on vaccine acceptability of the COVID-19 vaccine were conducted in various countries [14,15,16,17,18,19,20,29,30,31,32,33,34,35,36,37,38]. In Europe, it has been reported that the prevalence of vaccine acceptability was approximately from 60% to 80% [14,29,30,31,32,33,34,35,36]. Furthermore, a previous study in the United States, in which the same questionnaire was used, reported that as of December 2020, 56% of U.S. citizens stated they were somewhat or very likely to get vaccinated for COVID-19 [18]. Another study in the United States reported that vaccination intent for the COVID-19 vaccine increased overall from September 2020 (61.9%) to December 2020 (68.0%) [37]. A global survey of 19 countries showed that 71.5% of the participants responded that they would receive a vaccine if it was proven to be safe and effective [20]. Our results are similar to these percentages, although it is difficult to make a simple comparison between our study and previous ones because of the differences in how the questionnaires were administered and the timing of the surveys. Although Japan is one of the countries with the lowest vaccine confidence indexes in the world [22], regarding COVID-19 vaccine acceptance, there may not be a large difference between Japan and other countries. Nevertheless, further improvement of vaccine acceptance may be needed, as high vaccination coverage is necessary to prevent epidemics [39].

Vaccinations are widely recognized as one of the most effective preventive measures in public health [40]. However, in recent years, vaccine hesitancy among not only citizens, but also medical professionals, has become a problem [12,41,42]. Vaccine hesitancy varies across time, place, and type of vaccine, and is influenced by a variety of factors [12]. Therefore, it is necessary to assess vaccine acceptance of the COVID-19 vaccine and the factors that influence it in each region, in order to plan educational activities to increase acceptance of the vaccine. Previous studies conducted outside of Japan reported that various factors, such as sociodemographic factors, attitude and beliefs regarding COVID-19 infection and vaccination, and political views, influence decision-making of vaccine acceptance [14,15,16,17,18,19,20,29,30,31,32,33,34,35,36,37,38]. Our results show that vaccine acceptance in Japan was lower among several sociodemographic groups, such as women, adults aged 20–49 years, and those with low-income levels, which coincided with many previous studies [14,15,16,17,18,19,20,31,32,33,34,35,36,37]. To increase COVID-19 vaccine coverage in Japan, it may be important to ensure vaccination among these populations with low vaccine acceptance. Several attitudes and beliefs regarding COVID-19 infection and COVID-19 vaccination were also associated with vaccine acceptance, as reported in some previous studies [15,17,19,31,33,34,35]. Notably, these health beliefs, such as perceived susceptibility to the disease, and perceived severity of the disease, can be modified by interventions [43,44]. Therefore, interventions targeted at modifying such health beliefs about COVID-19 may lead to improved vaccination rates. The item with the highest OR for vaccine acceptance was ‘perceived effectiveness of the COVID-19 vaccine’, as reported in some previous studies [15,17]. Additionally, the item with the second highest OR was ‘willingness to protect others by getting oneself vaccinated’, which was also reported to be one of the important factors associated with COVID-19 vaccine acceptance in a previous study [19], and 489 participants had a low level of willingness to protect others by getting oneself vaccinated. This may suggest that a certain number of people want to be “freeloaders” who benefit from the indirect protection provided by the vaccination of other people, without getting vaccinated themselves [45]. Therefore, trying to change people’s perceptions of the effectiveness of the COVID-19 vaccine, and increasing awareness to willingly protect others by getting oneself vaccinated, may be important in promoting vaccine acceptance.

The strengths of our study include the large sample size and the selection of participants from all regions of Japan using quota sampling. However, the limitations of this study need to be considered as well. Firstly, participants were recruited from a single Internet research company. The latest White Paper 2020 issued by the Japanese government has reported that regular Internet users tend to have higher incomes than non-users [46], suggesting that the incomes of study participants are higher than the average Japanese citizen. Furthermore, results of this study, such as the proportion of participants with a high likelihood of getting a COVID-19 vaccine, may have been overestimated due to selection bias. Secondly, this study did not evaluate some psychological factors that may be associated with vaccine hesitancy, such as individuals’ engagement in extensive information searching [28]. Thirdly, because the acceptance of COVID-19 changes over time [12,18], acceptance of the COVID-19 vaccine when vaccination begins in Japan may differ. Despite these limitations, to the best of our knowledge, this is the first study to clarify the current COVID-19 vaccine acceptance and hesitancy situation in Japan and factors associated with such issues.

## 5. Conclusions

In conclusion, the self-reported likelihood of getting a COVID-19 vaccine before the start of vaccination in Japan was 62.1%. Our results indicate that vaccine acceptance was lower among several sociodemographic groups, such as women, adults aged 20–49 years, and those with low-income levels. Trying to improve people’s trust in the effectiveness of the COVID-19 vaccine and attempting to increase willingness among individuals to protect others by getting oneself vaccinated may be the key to promoting vaccine acceptance. These results may be useful in the planning of educational activities to increase the acceptance of the COVID-19 vaccine.

## Figures and Tables

**Table 1:** Participants’ characteristics.

Variables	Total	Participants Highly Likely to Get a COVID-19 Vaccine ^1^		Participants Unlikely to Get a COVID-19 Vaccine		*p*-Value ^4^
	*N* = 2956	*N* = 1836		*N* = 1120		
		62.1%		37.9%		
	*n*	*n* (%)		*n* (%)		
Sex						<0.001
Men	1458	991	(68.0)	467	(32.0)	
Women	1498	845	(56.4)	653	(43.6)	
Age						<0.001
20–49 years	1416	772	(54.5)	644	(45.5)	
50–64 years	761	484	(63.6)	277	(36.4)	
≥65 years	779	580	(74.5)	199	(25.5)	
Underlying diseases ^2^						<0.001
Yes	830	594	(71.6)	236	(28.4)	
No	2126	1242	(58.4)	884	(41.6)	
Marital status						<0.001
Married	1721	1138	(66.1)	583	(33.9)	
Not married	1235	698	(56.5)	537	(43.5)	
Employment status						0.617
Working	1788	1117	(62.5)	671	(37.5)	
Not working	1168	719	(61.6)	449	(38.4)	
Residential area						0.597
Tokyo metropolitan area ^3^	925	581	(62.8)	344	(37.2)	
Other	2031	1255	(61.8)	776	(38.2)	
Living arrangement						0.178
Alone	534	318	(59.6)	216	(40.4)	
With other	2422	1518	(62.7)	904	(37.3)	
Educational attainment						0.025
University graduate or above	1556	996	(64.0)	560	(36.0)	
Below University graduate level	1400	840	(60.0)	560	(40.0)	
Annual personal income						<0.001
<2 million yen (approximately 19,000 USD)	1420	790	(55.6)	630	(44.4)	
2–4 million yen (19,000–38,000)	765	517	(67.6)	248	(32.4)	
4–6 million yen (38,000–57,000)	437	299	(68.4)	138	(31.6)	
≥6 million yen or more (57,000–)	334	230	(68.9)	104	(31.1)	

^1^ We asked participants to indicate how likely they were to get vaccinated for coronavirus disease 2019 (COVID-19) once a vaccine is available to the public, and participants responded with 5 options (very unlikely, somewhat unlikely, somewhat likely, very likely, unsure). When a participant responded “very likely” or “somewhat likely”, it was determined that the participant had a high likelihood of getting a COVID-19 vaccine. ^2^ Underlying diseases included heart disease, respiratory disease, kidney disease, diabetes, and hypertension. ^3^ Tokyo metropolitan area included Tokyo, Kanagawa, Saitama, and Chiba prefectures. ^4^ *p*-value was calculated using the chi-square test.

**Table 2:** Attitudes and beliefs of participants regarding coronavirus disease 2019 (COVID-19) infection and COVID-19 vaccination.

Variables	Total	Participants Highly Likely to Get a COVID-19 Vaccine ^1^		Participants Unlikely to Get a COVID-19 Vaccine		*p*-Value ^3^
	*N* = 2956	*N* = 1836		*N* = 1120		
		62.1%		37.9%		
	*n*	*n* (%)		*n* (%)		
Perceived likelihood of becoming infected with COVID-19 in the future ^2^						0.001
Low	994	601	(60.5)	393	(39.5)	
Middle	1147	684	(59.6)	463	(40.4)	
High	815	551	(67.6)	264	(32.4)	
Perceived severity of a COVID-19 infection						<0.001
Low	840	476	(56.7)	364	(43.3)	
Middle	1025	600	(58.5)	425	(41.5)	
High	1091	760	(69.7)	331	(30.3)	
Perceived effectiveness of a COVID-19 vaccine						<0.001
Low	298	72	(24.2)	226	(75.8)	
Middle	608	230	(37.8)	378	(62.2)	
High	2050	1534	(74.8)	516	(25.2)	
Willingness to protect others by getting oneself vaccinated						<0.001
Low	489	226	(46.2)	263	(53.8)	
Middle	843	374	(44.4)	469	(55.6)	
High	1624	1236	(76.1)	388	(23.9)	
Significance of identified factors influencing respondents’ decision-making regarding vaccination						
Safety of vaccine						<0.001
Low	193	98	(50.8)	95	(49.2)	
Middle	472	261	(55.3)	211	(44.7)	
High	2291	1477	(64.5)	814	(35.5)	
Vaccination accessibility						<0.001
Low	320	156	(48.8)	164	(51.3)	
Middle	653	315	(48.2)	338	(51.8)	
High	1983	1365	(68.8)	618	(31.2)	
Doctor’s recommendation						<0.001
Low	522	294	(56.3)	228	(43.7)	
Middle	882	463	(52.5)	419	(47.5)	
High	1552	1079	(69.5)	473	(30.5)	

^1^ We asked participants to indicate how likely they were to get vaccinated for COVID-19 once a vaccine is available to the public, and participants responded with one of 5 options (very unlikely, somewhat unlikely, somewhat likely, very likely, unsure). When a participant responded “very likely” or “somewhat likely”, it was determined that the participant had a high likelihood of getting a COVID-19 vaccine. ^2^ Participants responded using a 7-point scale, and we coded each attitude and belief variable so that higher values indicate greater levels of that construct. When a participant responded with 1–3, 4, or 5–7 on the scale, the level of that construct was defined as low, middle, or high, respectively. ^3^ *p*-value was calculated using the chi-square test.

**Table 3:** Individual factors associated with high likelihood of getting a COVID-19 vaccine.

Variables		Model 1 ^1^			Model 2 ^2^		
	*n*	Odds Ratio	95% Confidence Interval	*p*-Value	Odds Ratio	95% Confidence Interval	*p*-Value
Sex:							
Men	1458	1.00			1.00		
Women	1498	0.74	(0.62–0.88)	0.001	0.67	(0.55–0.83)	<0.001
Age							
20–49 years	1416	0.45	(0.35–0.56)	<0.001	0.59	(0.45–0.77)	<0.001
50–64 years	761	0.60	(0.47–0.76)	<0.001	0.71	(0.54–0.94)	0.014
≥65 years	779	1.00			1.00		
Underlying diseases ^3^							
Yes	830	1.35	(1.12–1.63)	0.002	1.32	(1.06–1.65)	0.015
No	2126	1.00			1.00		
Marital status							
Married	1721	1.24	(1.02–1.50)	0.032	1.13	(0.91–1.41)	0.278
Not married	1235	1.00			1.00		
Employment status:							
Working	1788	1.08	(0.88–1.32)	0.476	1.08	(0.86–1.36)	0.492
Not working	1168	1.00			1.00		
Residential area:							
Tokyo metropolitan area ^4^	925	1.01	(0.85–1.19)	0.921	1.01	(0.83–1.22)	0.954
Other	2031	1.00			1.00		
Living arrangement:							
Alone	534	1.00			1.00		
With other	2422	1.08	(0.86–1.36)	0.521	1.08	(0.83–1.40)	0.586
Educational attainment:							
University graduate level or above	1556	1.15	(0.98–1.36)	0.092	1.04	(0.86–1.26)	0.679
Below University graduate level	1400	1.00			1.00		
Annual personal income							
<2 million yen (approximately 19,000 USD)	1420	0.71	(0.52–0.97)	0.033	0.67	(0.47–0.95)	0.026
2–4 million yen (19,000–38,000)	765	1.04	(0.77–1.40)	0.794	0.93	(0.67–1.31)	0.695
4–6 million yen (38,000–57,000)	437	1.03	(0.75–1.41)	0.846	1.00	(0.70–1.43)	0.997
≥6 million yen or more (57,000–)	334	1.00			1.00		
Perceived likelihood of becoming infected with COVID-19 in the future ^5^							
Low	994				1.00		
Middle	1147				1.04	(0.84–1.30)	0.692
High	815				1.58	(1.23–2.02)	<0.001
Perceived severity of a COVID-19 infection							
Low	840				1.00		
Middle	1025				1.18	(0.94–1.49)	0.149
High	1091				1.31	(1.02–1.68)	0.033
Perceived effectiveness of a COVID-19 vaccine							
Low	298				1.00		
Middle	608				2.39	(1.69–3.39)	<0.001
High	2050				9.15	(6.69–12.51)	<0.001
Willingness to protect others by getting oneself vaccinated							
Low	489				1.00		
Middle	843				1.37	(1.05–1.78)	0.022
High	1624				3.51	(2.75–4.48)	<0.001
Significance of identified factors influencing respondents’ decision-making regarding vaccination							
Safety of vaccine							
Low	193				1.00		
Middle	472				1.48	(0.95–2.29)	0.082
High	2291				0.64	(0.43–0.95)	0.027
Vaccination accessibility							
Low	320				1.00		
Middle	653				1.21	(0.84–1.73)	0.303
High	1983				1.98	(1.42–2.75)	<0.001
Doctor’s recommendation							
Low	522				1.00		
Middle	882				0.89	(0.67–1.17)	0.395
High	1552				1.18	(0.90–1.54)	0.224

^1^ The independent variables were sex, age (20–49/50–64/65 years and older), underlying diseases, marital status, employment status, residential area, living arrangement, educational attainment, and annual personal income. ^2^ The independent variables were the factors in model 1 plus perceived likelihood of becoming infected with COVID-19 in the future, perceived severity of a COVID-19 infection, perceived effectiveness of a COVID-19 vaccine, willingness to protect others by getting vaccinated, and the significance of identified factors influencing respondents’ decision-making regarding vaccination (three items). ^3^ Underlying diseases included heart disease, respiratory disease, kidney disease, diabetes, and hypertension. ^4^ Tokyo metropolitan area included Tokyo, Kanagawa, Saitama, and Chiba prefecture. ^5^ Participants responded using a 7-point scale, and we coded each attitude and belief variable so that higher values indicate greater levels of that construct. When a participant responded with 1–3, 4, or 5–7 on the scale, the level of that construct was defined as low, middle, or high, respectively.

## Data Availability

The data are available under reasonable request to the corresponding author.

## Supplementary Materials

The following are available online at https://www.mdpi.com/2076-393X/9/3/210/s1, Actual questions and response options translated from Japanese to English.
