# Supplementary material for: Acceptance of a COVID-19 Vaccine in Japan during the COVID-19 Pandemic
Source: Vaccines (Basel). 2021 Mar 3;9(3):210. doi: 10.3390/vaccines9030210 (PMC8002097; doi:10.3390/vaccines9030210)
Supplement: Supplementary file 1 [file vaccines-09-00210-s001.pdf]

Supplementary Material: Actual questions and response options translated from Japanese to English

Variable	Questions	Response options
Assessment of participants' likelihood of getting a COVID-19 vaccine [18]	How likely are you to get vaccinated for coronavirus once a vaccine is available to the public?	1.very unlikely 2. somewhat unlikely 3. somewhat likely 4. very likely 5. unsure
Perceived likelihood of becoming infected with COVID-19 in the future [26,27]	What do you consider to be your own probability of getting infected with COVID-19?	Extremely unlikely [1] [2] [3] [4] [5] [6] [7] Extremely likely
Perceived severity of a COVID-19 infection [26,27]	How severe would contracting COVID-19 be for you (how seriously ill do you think you will be)?	Not severe [1] [2] [3] [4] [5] [6] [7] Very severe
Perceived effectiveness of a COVID-19 vaccine [26,27]	I believe a vaccine can help control the spread of COVID-19	Strongly disagree [1] [2] [3] [4] [5] [6] [7] Strongly agree
Willingness to protect other by getting oneself vaccinated [26,27]	When everyone else is vaccinated against COVID-19, then I don't have to get vaccinated	Strongly agree [1] [2] [3] [4] [5] [6] [7] Strongly disagree
Significance of identified factors influencing respondents' decision-making regarding vaccination [26,27]	If a COVID-19 vaccine is made available in my country, my decision of whether or not to get vaccinated would depend on:	-
Safety of vaccines [26,27]	Whether the vaccine has been in use for a long time with no serious side-effects	Not at all [1] [2] [3] [4] [5] [6] [7] Very much so
Vaccine accessibility [26,27]	How easy it is to get the vaccine	Not at all [1] [2] [3] [4] [5] [6] [7] Very much so
Doctor's recommendation [26,27]	Recommendation from my family doctor	Not at all [1] [2] [3] [4] [5] [6] [7] Very much so

References:

18. Szilagyi, P.G.; Thomas, K.; Shah, M.D.; Vizueta, N.; Cui, Y.; Vangala, S.; Kapteyn, A. National Trends in the US Public's Likelihood of Getting a COVID-19 Vaccine-April 1 to December 8, 2020. JAMA 2020, 10.1001/jama.2020.26419, doi:10.1001/jama.2020.26419.

26. Betsch, C.; Wieler, L.H.; Habersaat, K.; COSMO group. Monitoring behavioural insights related to COVID-19. Lancet 2020, 395, 1255-1256, doi:10.1016/S0140-6736(20)30729-7.

27. World Health Organization regional office for Europe. COVID-19 Snapshot MOnitoring (COSMO). Available online: https://www.psycharchives.org/handle/20.500.12034/2397 (accessed on 28 January 2021).
